# Imaging mass cytometry reveals the prominent role of myeloid cells at the maternal-fetal interface

**DOI:** 10.1016/j.isci.2022.104648

**Published:** 2022-06-20

**Authors:** Juliette Krop, Anita van der Zwan, Marieke E. Ijsselsteijn, Hanneke Kapsenberg, Sietse J. Luk, Sanne H. Hendriks, Carin van der Keur, Lotte J. Verleng, Antonis Somarakis, Lotte van der Meeren, Geert Haasnoot, Manon Bos, Noel F.C.C. de Miranda, Susana M. Chuva de Sousa Lopes, Marie-Louise P. van der Hoorn, Frits Koning, Frans H.J. Claas, Sebastiaan Heidt, Michael Eikmans

**Affiliations:** 1 Department of Immunology, Leiden University Medical Center, Leiden, the Netherlands; 2 Department of Pathology, Leiden University Medical Center, Leiden, the Netherlands; 3 Department of Haematology, Leiden University Medical Center, Leiden, the Netherlands; 4 Division of Image Processing, Leiden University Medical Center, Leiden, the Netherlands; 5 Department of Pathology, University Medical Center Utrecht, Utrecht, the Netherlands; 6 Department of Gynecology and Obstetrics, Leiden University Medical Center, Leiden, the Netherlands; 7 Department of Anatomy and Embryology, Leiden University Medical Center, Leiden, the Netherlands

**Keywords:** Biological sciences, Immunology, Biotechnology, Biological sciences research methodologies

## Abstract

Although the immunological complexity of the maternal-fetal interface is well appreciated, the actual interaction of maternal immune cells and fetal trophoblasts is insufficiently understood. To comprehend the composition and spatial orientation of maternal immune cells and fetal extravillous trophoblasts, we applied imaging mass cytometry on decidua basalis of the three trimesters of healthy pregnancy. Within all trimesters, we observed considerably higher frequencies of myeloid cells in the decidua than is seen with single-cell suspension techniques. Moreover, they were the most pronounced cell type in the microenvironment of other decidual cells. In first trimester, HLA-DR^-^ macrophages represented the most abundant myeloid subcluster and these cells were frequently observed in the vicinity of trophoblasts. At term, HLA-DR^+^ macrophage subclusters were abundantly present and frequently observed in the microenvironment of T cells. Taken together, our results highlight the dynamic role of myeloid cells at the human maternal-fetal interface throughout gestation.

## Highlights

• Frequency of myeloid cells is underestimated after tissue digestion
• Myeloid cells could support NK cells with proper trophoblast invasion
• Myeloid cells are dynamic in their role throughout gestation

## Graphical abstract

**Figure:**
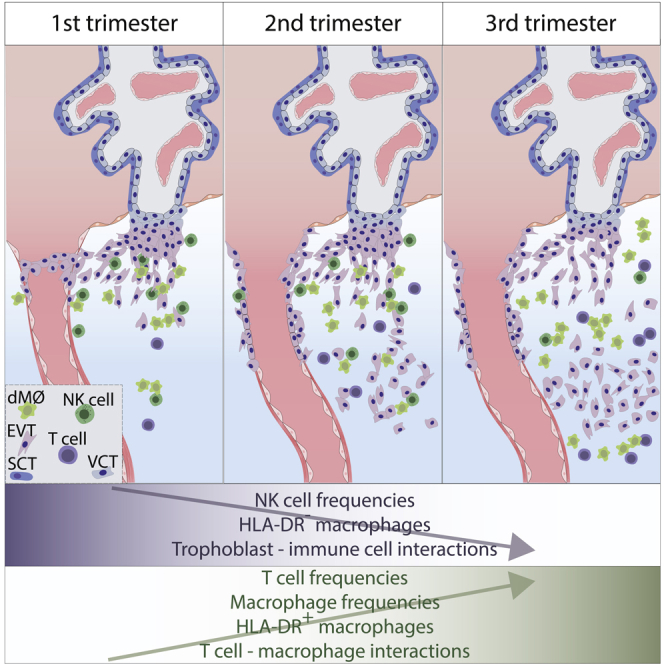

## Introduction

The placenta is essential in providing nutrients and oxygen to the developing fetus during pregnancy. During early placentation, fetal trophoblasts invade into the maternal endometrium. When this invasion occurs, fetal trophoblasts encounter maternal immune cells which may recognize the fetal cells and affect invasion. Disturbed trophoblast invasion likely occurs in several pregnancy complications and is associated with alterations in the composition of the maternal immune cells at the decidua (Yeh et al., 2013; Dunk et al., 2019; Guo et al., 2021; Yang et al., 2019). Because pregnancy complications can be divergent in their time of onset, it is important to analyze the maternal decidual immune cell composition throughout gestation. However, before studying pregnancy complications, it is essential to gain understanding in how trophoblasts and immune cells interact with one another at different trimesters during healthy pregnancy.

Most research on phenotyping of decidual maternal immune cells during gestation has been performed using suspension techniques. Hereby, the tissue is mashed over a filter, usually followed by an enzymatic digestion step. Based on the results of single cell suspension techniques the most prominent maternal immune cells in the first trimester at the maternal-fetal interface have been described as NK cells (∼60%), followed by macrophages (∼20%), and T cells (∼10%) (Gomez-Lopez et al., 2010; Van Der Zwan et al., 2020). Later in gestation the NK cells decrease and T cells increase in frequency, whereas macrophage proportions remain constant, suggesting altering functions for NK cells and T cells at different trimesters (Vince et al., 1990; Starkey et al., 1988; Van Der Zwan et al., 2020; Williams et al., 2009). Based on these observations extensive phenotyping using single cell suspension techniques of decidual maternal immune cells has mainly focused on NK cells and T cells using specifically designed panels, largely neglecting the myeloid compartment (Huhn et al., 2020; Van Der Zwan et al., 2020). Besides phenotypic analysis, the functional role of decidual maternal immune cells has been studied frequently by using *in vitro* assays or by using prediction algorithms based on suspension scRNA-seq data (Vento-Tormo et al., 2018). In these types of studies, however, information on spatial orientation of the cells is missing: if particular cells do not encounter each other *in situ*, effects observed in culture assays with these cell types might not be relevant.

*In situ* techniques, such as immunohistochemistry, have been scarcely used to determine phenotypes, or to quantify the distribution of the major immune cell lineages over different trimesters (Williams et al., 2009; Bulmer et al., 1991; Haller et al., 1993). Nonetheless, *in situ* techniques can provide a more accurate quantification, as there is no specific cell loss because of the isolation process. Furthermore, it provides the spatial information, which can be used for designing relevant cell culture assays. Imaging mass cytometry (IMC) provides the possibility for in-depth studies on immune cell interactions in the decidua.

Macrophages and other myeloid cells appear to be stably present during gestation and are an essential part of the intricate immune network at the maternal-fetal interface (Van Der Zwan et al., 2020). Phenotypically, macrophage populations are often subdivided into two subpopulations, namely M1 and M2 (Jiang and Wang, 2020; Ning et al., 2016; Van Der Zwan et al., 2020). However, this terminology is misrepresenting the broader spectrum of macrophage variation. Macrophages have a high plasticity, as their phenotype is continuously being altered in response to environmental cues (Martinez and Gordon, 2014; Gordon and Plüddemann, 2017; Wu et al., 2021). In-depth phenotyping could help visualize the dynamic changes in the different subtypes and maturation/activation stages of macrophages. Because of the near-absence of B cells and dendritic cells at the maternal-fetal interface, macrophages represent the main type of antigen-presenting cells in the decidua (Van Der Zwan et al., 2020). Decidual macrophages have generally been described to express HLA-DR (Ning et al., 2016; Vince et al., 1990; Bulmer and Johnson, 1984). Therefore, they likely play a crucial role in antigen presentation to CD4^+^T cells and may thereby contribute to the establishment of maternal-fetal immune tolerance, as well as regular immune surveillance. Macrophages have also been described to secrete a wide range of growth factors and cytokines, which suggests that they may have important functions throughout gestation, including during blastocyst implantation, trophoblast invasion, and for tissue homeostasis (Renaud and Graham, 2008; Reister et al., 1999; Svensson-Arvelund et al., 2015).

Here, we applied IMC on the human decidua to define the spatial orientation of immune cells in relation to each other and to fetal trophoblasts cells. First, we phenotypically determined the immune cell clusters present in the decidua and assessed their distribution over the different trimesters. Next, we determined the microenvironment profiles of both immune cells and trophoblast cells. Hereby, we focused on the myeloid cell compartment to gain knowledge of its potential role throughout different trimesters. Overall, our 42-marker IMC panel provides a baseline for immune cell phenotype and distribution, as well as their direct microenvironment at the decidua basalis during first, second, and third trimester.

## Results

### Differential decidual immune cell frequencies in tissue sections versus digested tissue suspensions

**Figure 1:**
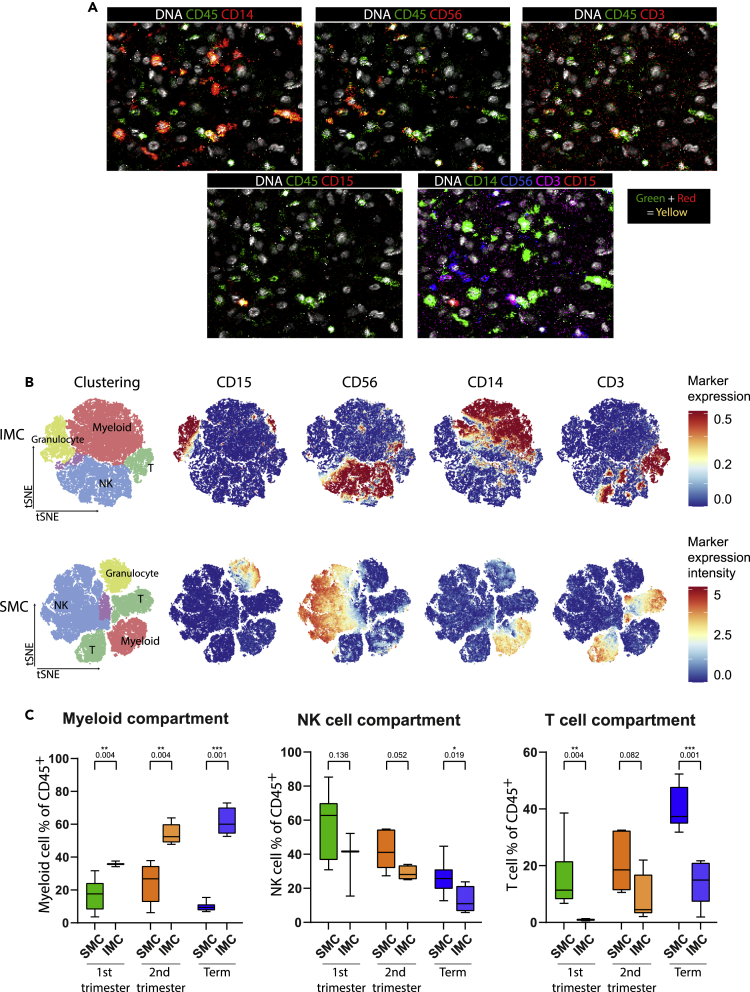
Comparison of major immune cell lineage frequencies at the maternal-fetal interface by IMC and SMC (A) Raw IMC data show that all major immune lineages can be distinguished. Using a mask, the IMC data is segmented in single cells with DNA as primary object. (B) tSNE of the major immune cell lineages within the CD45^+^ compartment of IMC and SMC by automatic clustering using 17 overlapping markers (Table 2). For visualization but not for quantification, SMC is randomly down sampled to the same number of cells as IMC (54.351 cells). For IMC, the marker expression is binarized from 0 to 1, meaning one cell with 0.5 expression has 50% of pixels in the cell being positive for the marker. Whereas in the SMC data the marker expression visualizes the intensity of a marker (arcsin transformed to 5) such as bright or dim expression of a marker. The red cluster is the myeloid cluster, green the T cell cluster, blue the NK cell cluster, yellow the granulocyte cluster and purple non-defined CD45^+^ cells (debris and B cells). (C) Comparison between SMC and IMC of the frequency (as percentage of CD45^+^ cells) of the myeloid cell- (without granulocytes), NK cell- and T cell compartment at all three trimesters (first trimester SMC n = 12, IMC n = 3; second trimester SMC n = 6, IMC n = 5; term SMC n = 9, IMC n = 5). Myeloid cells where significantly higher frequent in IMC than SMC at all three trimesters (first trimester: median 17.6 versus 35.8%, p = 0.004; second trimester: median 26.8 versus 52.5%, p = 0.004; Term: median 9.4 versus 60%, p= 0.001). NK cells showed a trend toward a decreased frequency in IMC compared to SMC, this decrease was significant at term (first trimester: median 62.8 versus 41.6%, p = 0.136; second trimester: median 41.1 versus 28.1%, p = 0.052; Term: median 25.7 versus 11%, p= 0.019). T cells showed significantly decreased frequency by IMC compared to SMC in first trimester and term (first trimester: median 11.4 versus 0.9%, p = 0.004; second trimester: median 18.5 versus 4.5%, p = 0.082; Term: median 37.3 versus 14.9%, p= 0.001). Data are represented as Min to Max boxplots, Mann-Whitney test, ∗p< 0.05; ∗∗p< 0.01; ∗∗∗p< 0.001. IMC, imaging mass cytometry; SMC, suspension mass cytometry; tSNE, t-distributed stochastic neighbor embedding; HSNE, Hierarchical Stochastic Neighbor Embedding.

For analysis of frequencies and visualization of immune cells in their spatial context, we designed and optimized a 42-marker IMC panel for application on human decidual samples (Table 2). In our previous work, we visualized the immune cell compartment of the decidua basalis at first, second, and third trimester using suspension mass cytometry (SMC) (Van Der Zwan et al., 2020). We compared results of SMC and IMC to determine the possible effect of sample processing on cell frequencies in the suspension technique. Frequencies of the major immune cell populations within the CD45^+^ cell compartment, using the 17 overlapping markers in both panels, were evaluated (Figures 1, S1, and S2, and Table 2). The four major immune cell lineage markers, CD14, CD56, CD3 and CD15 present in both panels are visualized in Figure 1A for IMC. Using a cell mask to segment all cells into single cells the IMC data could be quantified similarly to SMC data using tSNE (Figure 1B).

**Table 1:** Patient characteristics and measurement details

	SampleID	Trimester	Gestational age (weeks + days)	Delivery mode	Maternal age	ROIs measured	area measured (mm^2^)^a^	# of immune cells detected/analyzed	# of trophoblasts detected/analyzed	Ratio immune cells/trophoblasts
1	2307	First	10	Suction curettage	unknown	5	4.62	5926	5225	1.134
2	2317	First	11	Suction curettage	unknown	5	6.71	7326	6680	1.097
3	2331	First	10	Suction curettage	unknown	7	4.5	3939	3612	1.091
4	2177	Second	21	Surgical abortion	unknown	8	4.76	1511	6243	0.242
5	2268	Second	18	Surgical abortion	unknown	8	6.87	2974	4739	0.628
6	2280	Second	18	Surgical abortion	unknown	7	3.95	2983	2898	1.029
7	2297	Second	16	Surgical abortion	unknown	7	6.87	10,190	4390	2.321
8	2324	Second	15	Surgical abortion	unknown	7	5.82	6489	5202	1.247
9	2366	Term	39	primary c-section	30	6	2.25	1041	489	2.129
10	2374	Term	39	primary c-section	32	12	4.85	2514	1003	2.506
11	2394	Term	41 + 1	spontaneous	36	9	8.93	4244	2772	1.531
12	2427	Term	42 + 1	secondary c-section	37	8	6.28	1912	2819	0.678
13	2398	Term	41 + 4	spontaneous	32	9	6.7	1820	5856	0.311

a Area of region of interest (ROI) including tissue and background.

**Table 2:** Imaging mass cytometry antibody panel

	Target	Clone	RRID	Metal	Incubation time	Temperature	Dilution
1	Pan-Keratin	C11 and AE1/AE3	N/A	^106^Pd	Overnight	4°C	50
2	Collagen I	EPR7785	N/A	^115^In	Overnight	4°C	50
3	**HLA-DR**	TAL 1B5	N/A	^141^Pr	5 h	RT	100
4	EGF-R	D38B1	N/A	^142^ND	Overnight	4°C	50
5	CD68	D4B9C	N/A	^143^ND	Overnight	4°C	100
6	**CD11b**	D6X1N	N/A	^144^ND	5 h	RT	100
7	**CD4**	EPR6855	AB_2864377	^145^ND	Indirect	4°C	50
8	**CD8α**	D8A8Y	N/A	^146^ND	5 h	RT	50
9	CD31	89C2	N/A	^147^Sm	Overnight	4°C	100
10	CD73	D7F9A	N/A	^148^ND	5 h	RT	100
11	**CD69**	EPR21814	AB_2891140	^149^Sm	Overnight	4°C	100
12	Granzyme B	D6E9W	N/A	^150^ND	5 h	RT	100
13	CD66b	G10F5	N/A	^151^Eu	5 h	RT	100
14	Ki-67	8D5	N/A	^152^Sm	Overnight	4°C	100
15	**CD3**	EP449E	N/A	^153^Eu	Overnight	4°C	50
16	TIM3	D5D5R(TM)	N/A	^154^Sm	5 h	RT	100
17	CD141	E7Y9P	N/A	^155^Gd	Overnight	4°C	50
18	NKG2A	LS-C165590	N/A	^156^Gd	5 h	RT	50
19	CD39	EPR20627	N/A	^157^Gd	5 h	RT	100
20	CD1c	EPR23189-196	AB_2884015	^158^Gd	5 h	RT	50
21	FOXp3	D608R	N/A	^159^Tb	Overnight	4°C	50
22	**PD-1**	D4W2J	N/A	^160^Gd	5 h	RT	50
23	DC-SIGN	NBP1-77284	N/A	^161^Dy	Overnight	4°C	50
24	IDO	D5J4E(TM)	N/A	^162^Dy	Overnight	4°C	100
25	**CD14**	D7A2T	N/A	^163^Dy	5 h	RT	100
26	CD204	J5HTR3	N/A	^164^Dy	5 h	RT	50
27	**CD45RO**	UCHL1	AB_2563752	^165^Ho	Overnight	4°C	100
28	D2-40	D2-40	N/A	^166^Er	Overnight	4°C	100
29	**CD56**	E7X9M	N/A	^167^Er	5 h	RT	100
30	**CD103**	EPR4166(2)	N/A	^168^Er	5 h	RT	50
31	**CD38**	EPR4106	AB_2864383	^169^Tm	Overnight	4°C	100
32	**CD45RA**	HI100	N/A	^170^Er	5 h	RT	100
33	**CD15**	BRA-4F1	N/A	^171^Yb	Overnight	4°C	100
34	Cleaved caspase-3	ASP175	N/A	^172^Yb	5 h	RT	100
35	**CD163**	EPR14643-36	N/A	^173^Yb	5 h	RT	50
36	CD7	EPR4242	AB_2889384	^174^Yb	5 h	RT	100
37	**CD45**	D9M8I	N/A	^175^Lu	5 h	RT	50
38	**CD11c**	EP1347Y	AB_2864379	^176^Yb	5 h	RT	100
39	Vimentin	D21H3	N/A	^194^Pt	Overnight	4°C	50
40	HLA-G	MEM-G2	N/A	^198^Pt	5 h	RT	100
41	αSMA	D4K9N	N/A	^209^Bi	5 h	RT	100
42	Βcatenin	D10A8	N/A	^89^Y	Overnight	4°C	100

In bold are the markers that are overlapping with the SMC panel.

To compare the IMC tissue approach with the suspension technique, the frequency of granulocytes, other myeloid cells (monocytes, macrophages, DCs), NK cells, and T cells were determined at first trimester, second trimester, and term. SMC only stains extracellularly located proteins while IMC also stains intracellular proteins. For that matter, decidual NK cells may express intracellular CD3 as observed in Figure 1B (Vento-Tormo et al., 2018). Kinetics for the majority of the cell types were comparable over time between IMC and SMC (Figures 1C and S2). The main difference observed was the high frequency of myeloid cells in IMC compared to SMC (1^st^ trimester: median 35.8% versus 17.6%, p = 0.004; 2^nd^ trimester: median 52.5% versus 26.8%, p = 0.004; Term: median 60% versus 9.4%, p = 0.001) (Figure 1C). Furthermore, we found a significant increase in IMC of the myeloid compartment represented 35.8%, 52.5%, 60% of the CD45^+^ cell compartment at first, second, and third trimester, respectively (first trimester versus term: p = 0.012). Consequently, frequencies of NK cells within the CD45^+^ compartment by IMC were lower compared to SMC at term (median 11% versus 25.7%, p = 0.019). A similar finding applied to T cells, where the frequency was significantly lower in first trimester and term (first trimester: median 0.9 versus 11.4%, p = 0.004; Term: median 14.9 versus 37.3%, p = 0.001) (Figure 1C). For NK cells determined by IMC at first, second, and third trimester the median frequency within the CD45^+^ compartment was 41.6%, 28.1%, and 11%, respectively and for T cells 0.9%, 4.5%, and 14.9%, respectively.

### *In situ* identification of 16 phenotypically distinct immune cell clusters present in the decidua basalis at different trimesters

**Figure 2:**
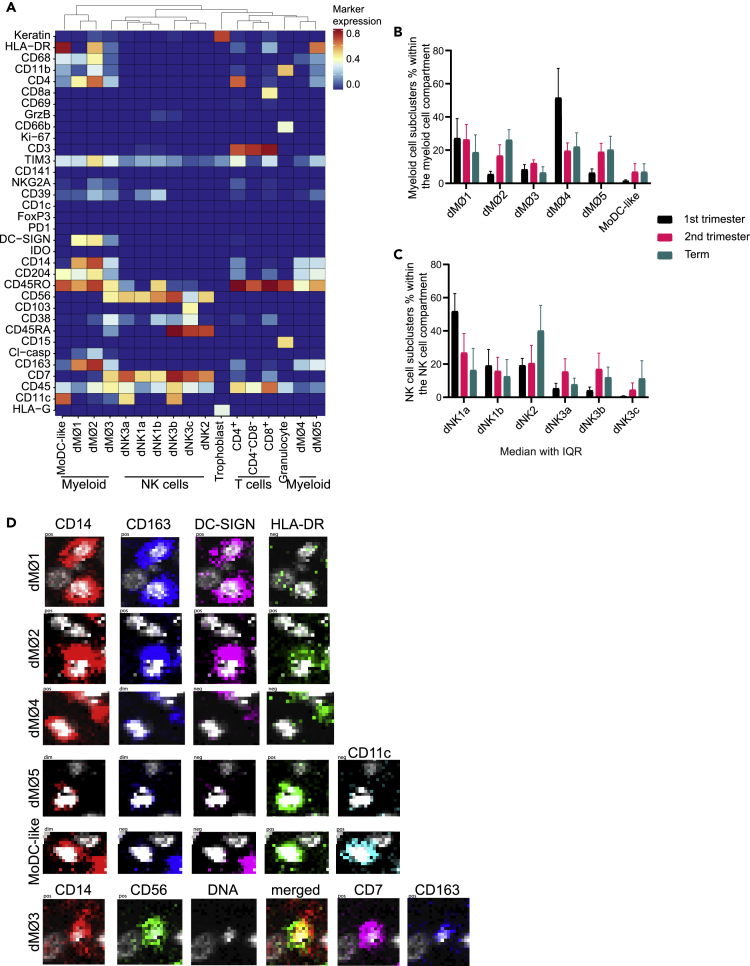
Identification of immune cell subclusters and trophoblasts at the maternal-fetal interface (A) All 16 identified subclusters of different immune cell lineages and trophoblasts of all trimesters are visualized in a heatmap showing marker expression (white to red) or absence thereof (blue) (13 samples, 92,855 cells). Cluster IDs are displayed at the bottom of the heatmap, marker names are on the left. (B) Myeloid cell subcluster frequencies are visualized within the myeloid compartment over the different trimesters. The overall distribution over the different subclusters is significantly different in first trimester (p<0.001), second trimester (p = 0.020), and term (p = 0.010). In first trimester DR^−^SIGN^+^ and DR^−^SIGN^-^ represent the most prominent subclusters. (C) NK cell subcluster frequencies are shown within the NK cell compartment over the different trimesters. The overall distribution over the different subclusters is significantly different in first trimester (p<0.001), second trimester (p = 0.027), but not at term (p = 0.080). At first trimester CD45RO^−^RA^-^ represents the most prominent subcluster. (D) Raw IMC data showing positive (pos), dim or negative (neg) expression of the markers with the biggest variation between the different myeloid cell subclusters. (B and C) Data are represented as medians with IQR, Friedman test per trimester.

**Figure 3:**
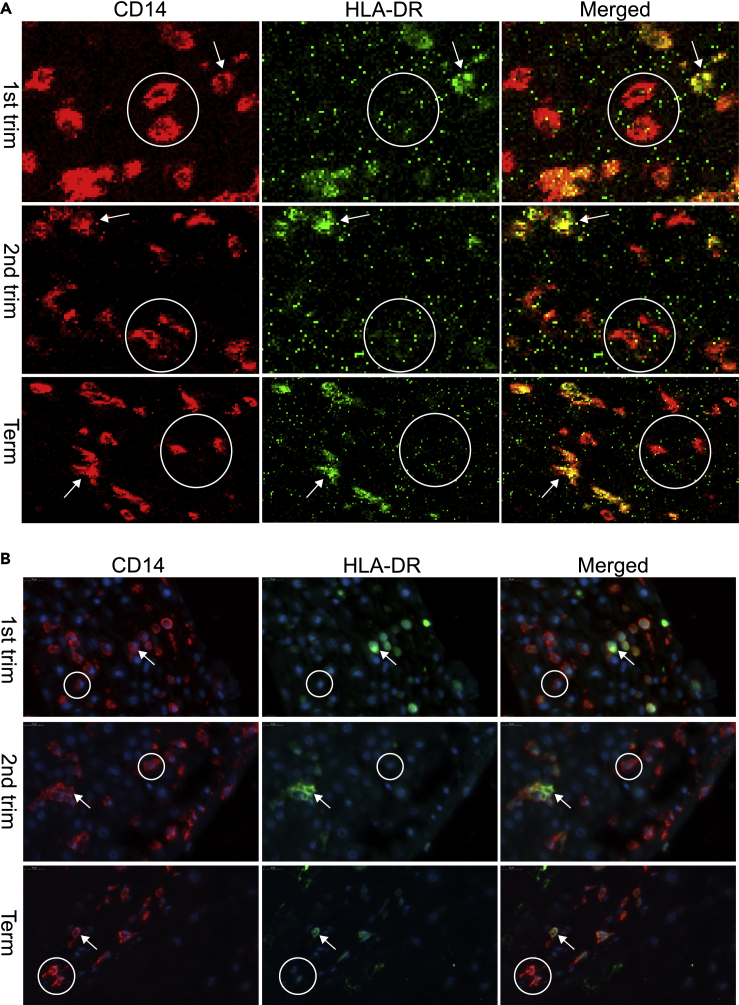
Verification of absence of HLA-DR on myeloid cell subclusters at the maternal-fetal interface by immunofluorescence (IF) staining (A) In all trimesters (and all samples), myeloid cells (CD14^+^) that lack HLA-DR expression (circle), and myeloid cells that do have HLA-DR expression (arrow) are observed. (B) Confirmation by IF that not all CD14^+^ cells in the decidua express HLA-DR (circle), while others do (arrow).

On basis of the immune cell markers (n = 33) of the complete IMC panel, we were able to identify 16 phenotypically different immune cell clusters (Figure 2A). These clusters were found in all samples, but were differently distributed over the trimesters (Figures 2B, 2C, and S3). Six phenotypically distinct subclusters were identified in the myeloid compartment by density-based clustering using Cytosplore (Figures 2B, 2D, and S3) (Höllt et al., 2016). Four of the six subclusters could clearly be distinguished from one another by their expression of HLA-DR and DC-SIGN, referred to as dMØ1, dMØ2, dMØ4 and dMØ5 (Figures 2B and 2D). Multiple other markers besides HLA-DR and DC-SIGN were differentially expressed between subclusters, as shown in Figures 2A and S3. Two of the HLA-DR^-^ subclusters (dMØ1 and dMØ4) were especially prominent in first trimester samples (Figure 2B). The two HLA-DR^-^ subclusters showed no detectable HLA-DR expression in the IMC analysis (Figure 3A). We validated the low expression or absence of HLA-DR on a subset of CD14^+^ cells by applying immunofluorescence (IF) staining using a secondary antibody for amplification of the signal intensity (Figure 3B). Despite this amplification, a subset of HLA-DR^-^ myeloid cells was still observed. Furthermore, we also found HLA-DR dim and HLA-DR negative myeloid cells in the SMC data (Van Der Zwan et al., 2020).

One myeloid subcluster (dMØ3) expressed both NK cell markers and myeloid markers, which has not previously been described in the decidua. Besides the myeloid markers CD14, CD68, and CD163, this population expressed NK cell markers CD56 and CD7 (Figures 2D and S3). To exclude an artifact of IMC, we cross-referenced our SCM data (Van Der Zwan et al., 2020), whereby we also found this cluster (Figure S4A). In further support of our finding, we also identified this cluster in an independent dataset previously published by Vento-Tormo et al. (2018) (Figure S4B). In addition, we found one myeloid subcluster (MoDC-like) that does not seem to express CD14, while expressing CD204, HLA-DR, and CD11c. Furthermore, we identified granulocytes, which were CD66b^+^CD15^+^. This subset was not included in further analysis.

T cells are known to be present in relatively low frequencies in the decidua. Based on our previous data (Van Der Zwan et al., 2020) we found that a reliably defined cluster consists of >100 cells and we therefore merged all T cells into three subclusters: CD4^+^T cells, CD8^+^T cells, and CD3^+^CD4^−^CD8^−^T cells.

We found 6 different NK cell subclusters. dNK1 were characterized by CD69 expression (dNK1a, dNK1b). The dNK2 subcluster was identified based on absence of CD69, CD11c or CD103 expression, as recently characterized by Vento-Tormo et al. (2018) (Figures 2A and S3). Finally, dNK3 were characterized by CD11c (dNK3a, dNK3b) or CD103 (dNK3c) expression. In concordance with previous literature the dNK1 subclusters were the most prominent dNK cells present in first trimester (Vento-Tormo et al., 2018), whereas in second trimester and term most dNK cell subclusters were distributed more equally (Figure 2D).

### Myeloid cells are the most prominent immune cells in the microenvironment of trophoblasts at all three trimesters

In addition to immune cells, we visualized trophoblasts *in situ* based on expression of Keratin and HLA-G (Figures 2A and S5A). At first trimester, trophoblasts were present in equal numbers to immune cells (Table 1). In second trimester and term there was more variation in the ratio of immune cells over trophoblasts between samples.

**Figure 4:**
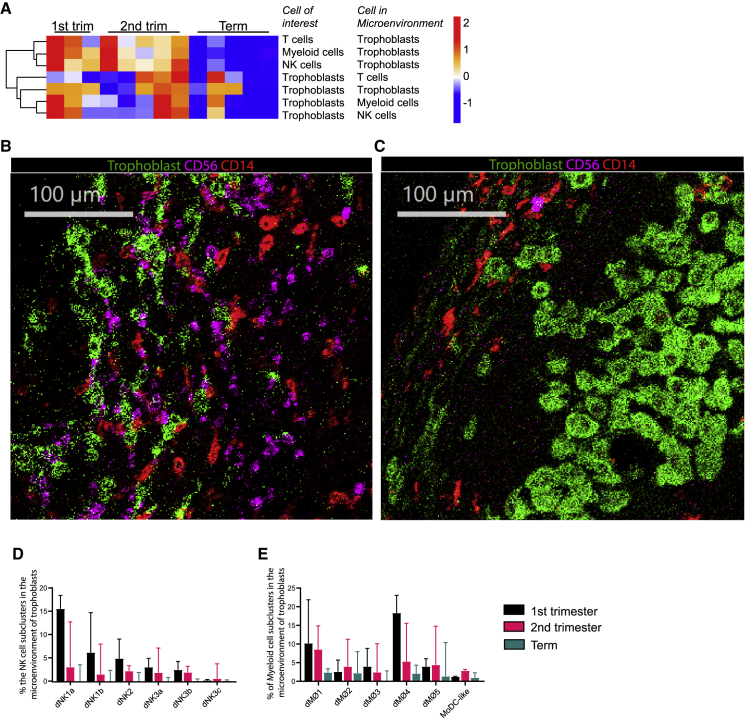
Microenvironment profile of trophoblasts (A) *Z* score hierarchical clustering heatmap visualization of trophoblast interactions with major immune cell lineages at first trimester, second trimester, and term. Orange/red indicates a higher amount of interactions and blue a lower amount of interactions than the mean of corrected microenvironment analysis frequency per row. (B and C) Visualization of myeloid cell (red) and NK cell (magenta) interactions with trophoblasts (green) in (B) first trimester and (C) term decidua. (D) Frequency of the six NK cell subclusters (of all immune cells and trophoblasts) that are within the microenvironment of trophoblasts. A Friedman test of first trimester samples indicates a differential distribution of the subclusters in the microenvironment of trophoblasts (p<0.001). A Kruskal-Wallis test of the three trimesters and subcluster dNK1a shows a significant decrease of dNK1a cells in the microenvironment of trophoblasts (Kruskal-Wallis p = 0.020; multiple comparisons first trimester versus term p = 0.033). (E) Frequency of the six myeloid subclusters (within both immune cell and trophoblast compartment) that are within the microenvironment of trophoblasts. A Friedman test of first trimester samples indicates a differential distribution of the subclusters in the microenvironment of trophoblasts (p<0.001), similarly for second trimester (p = 0.014), but not for term (p = 0.115). A Kruskal-Wallis test of the three trimesters and subcluster dMØ4 showed a significant decrease of dMØ4 cells in the microenvironment of trophoblasts (Kruskal-Wallis p = 0.014; multiple comparisons first trimester versus term p = 0.024). (B and C) Data are represented as medians with IQR.

Next, we systemically analyzed the microenvironment of trophoblasts and immune cells in the tissue within a 10-pixel (10 μm) radius and corrected for random co-localization based on cell frequencies (Figure S6). This approach is specifically oriented at identifying cells that might influence neighboring cells by direct contact and/or secreted products. When analyzing the microenvironment of trophoblasts, we observed a steady decrease in all immune cells in the vicinity of trophoblasts at term (Figure 4A and S7A), as can be observed in representative images of first trimester (Figure 4B) and at term (Figure 4C).

Because many studies have focused on the role of dNK cells in first trimester we used that information to confirm our microenvironment analysis methodology. The presence of dNK cells in the vicinity of trophoblasts during early invasion has been described to be important for proper trophoblast invasion and spiral artery remodeling (reviewed in (Jabrane-Ferrat and Siewiera, 2014)). Recently Vento-Tormo suggested that dNK1 specifically interact with trophoblasts (Vento-Tormo et al., 2018). Using our IMC data we could indeed confirm that at first trimester NK cell interactions with trophoblasts frequently occur, and specifically this is mediated by dNK1s (Figure 4D).

When focusing on myeloid subclusters in the trophoblast cell microenvironment, we observed at first trimester that myeloid cells were primarily represented by the two HLA-DR^-^ subclusters (dMØ1 and dMØ4) (Figure 4E). Interestingly, at term relatively low frequencies of myeloid cells were detected in the microenvironment of trophoblasts, despite the relative frequency of myeloid cells at term being higher than in first trimester (Figure S2B). This could be the result of deposition of fibrinoid encompassing trophoblast cells (Ds Goi et al., 2010). Indeed, we observed fibrinoid deposition to be increased in term decidua in both IMC and consecutive hematoxylin and eosin stained slides (Figure S5A). The fibinoid deposition stained positive for collagen IV, fibronectin and at some locations for pan-Laminin (Figure S5B). T cells were nearly absent in the trophoblast cell microenvironment at all trimesters (Figure S7).

### Myeloid cells represent the most dominant cell type in the microenvironment of other immune cells at all three trimesters

**Figure 5:**
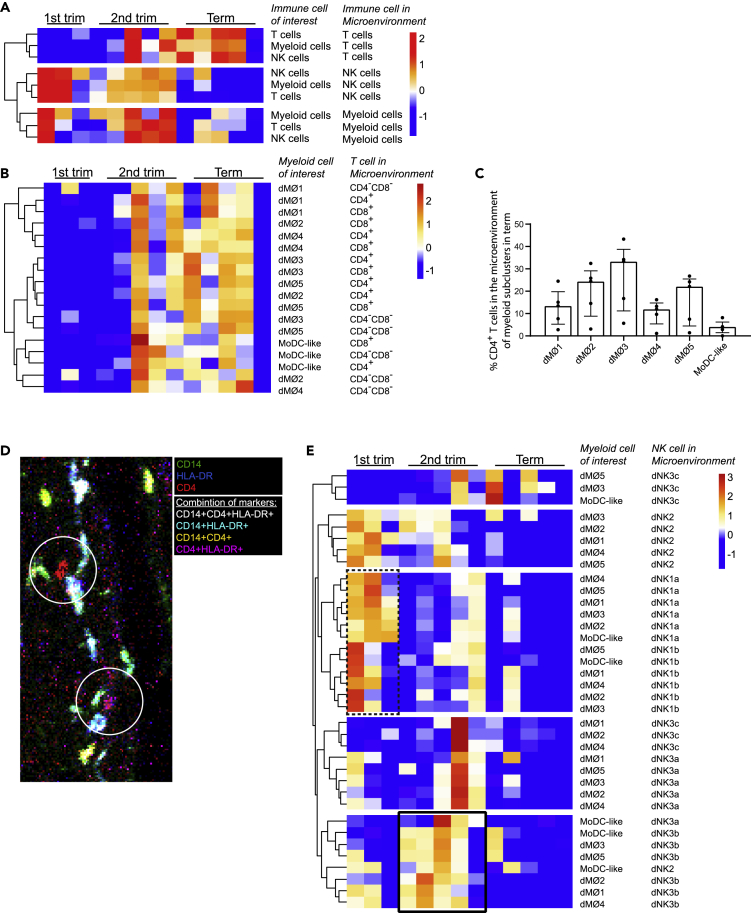
Microenvironment profile of immune cells In the *Z* score hierarchical clustering heatmaps, orange/red indicates a higher amount of interactions and blue a lower amount of interactions than the mean of corrected microenvironment analysis frequency per row. (A) Visualization of the changes between trimesters of major immune cell lineages with other major immune cell lineages in their microenvironment. (B) Visualization of the changes between trimesters of myeloid subclusters with T cells clusters in their microenvironment. (C) Frequency of the amount of CD4^+^T cells (of all immune cells and trophoblasts) that are in the microenvironment of the six myeloid cell subclusters at term. In the microenvironment of the six myeloid subclusters at term there is an unequal distribution CD4^+^T cells in their microenvironment (Friedman test p<0.001). (D) Visualization of HLA-DR^+^ (white and cyan) and HLA-DR^-^ (green and yellow) myeloid cells in the microenvironment of (circles) CD4^+^T cells (red and magenta) in a term placenta. (E) Visualization of the changes between trimesters of myeloid subclusters with NK cell subclusters in their microenvironment. The dotted box highlights that the most prominent NK cell subclusters present in the microenvironment of all six myeloid subclusters at the first trimester are dNK1a and dNK1b. The black box highlights that the most prominent NK cell subcluster in the microenvironment of all six myeloid subsets at the second trimester is the dNK3b subcluster.

The presence of the major immune cells in the microenvironment of other immune cells was also studied. Myeloid cells were the most abundantly present immune cell in the microenvironment of all other immune cells (Figure S7A, blue lines). The frequency of myeloid cells in the microenvironment of other immune cells remained relatively stable over time (Figure S7A), even though the relative cell frequency of myeloid cells increased during gestation (Figure S2). This indicates that we used an appropriate correction method for random co-localization based on cell frequencies. For further verification we used permutation testing and found that the interactions identified do not occur at random (Figure S7B). In contrast to the stable presence of myeloid cells in the microenvironment of other immune cells over time, we observed an increase of T cells in the microenvironment of other immune cells from first trimester to term, accompanied by a decrease of NK cells in the microenvironment of other immune cells (Figure 5A).

Next, we analyzed the subclusters within the microenvironment of the myeloid compartment. First, we observed a trend toward an increase in the amount of T cells in the microenvironment of myeloid cells from first trimester to term (Figures 5A and S7). This was both the case for CD4^+^T cells and CD8^+^T cells (Figure 5B). At term, there was an uneven distribution of T cells in the microenvironment of myeloid subclusters (Friedman test p = 0.001). Three HLA-DR^+^ macrophage subclusters (dMØ2, dMØ3, dMØ5) had a higher amount of CD4^+^T cells in their microenvironment at term than the HLA-DR^-^ macrophage subclusters (dMØ1, dMØ4) (Figure 5C). Figure 5D shows a representative image were HLA-DR^-^ macrophages (green and yellow) are less often in the microenvironment (circles) of CD4^+^T cells (red and magenta) than HLA-DR^+^ macrophages (white and cyan)

Lastly, we observed that the decrease of NK cells in the microenvironment of myeloid cells from first trimester to term could not be ascribed to one specific myeloid subcluster or NK cell subcluster (Figure 5E). In first trimester, the NK cell dNK1a and dNK1b subclusters were prominently present in the microenvironment of macrophages (Figure 5E, dashed box). In second trimester, the NK cell subset dNK3b became increasingly present in the microenvironment of all myeloid subclusters (Figure 5E, black box). At term, there was no particular NK subset highly present in the microenvironment of myeloid subclusters.

## Discussion

To comprehend the composition and spatial orientation of maternal immune cells and fetal extravillous trophoblasts, we applied in-depth immune profiling and microenvironment analysis on tissue sections of first trimester, second trimester, and term decidua from healthy pregnancies. We demonstrated that myeloid cells are present in high numbers in the decidua and that these cells are also highly abundant in the microenvironment of other cells. Furthermore, we showed that the distribution of different myeloid subclusters changes throughout the subsequent trimesters, which may go hand in hand with a functional change, as suggested by the microenvironment analysis.

Suspension techniques can be subject to bias because of incomplete tissue digestion, peripheral blood contamination and contamination by non-decidual tissue such as fetal villi and peripheral blood. In contrast to suspension techniques, we observed a higher frequency of macrophages throughout all three trimesters. Myeloid cells constituted ∼35% of the immune cell compartment in the decidua during first trimester and ∼60% during second and third trimester, as compared to ∼20% within all trimesters as determined using suspension techniques (Vince et al., 1990; Van Der Zwan et al., 2020; Gomez-Lopez et al., 2010). Our combined results of IMC and SMC suggest a selective loss of myeloid cells when using suspension techniques for phenotyping. Consequently, the importance of myeloid cells in the decidua during all trimesters in healthy pregnancy may previously have been underestimated. In addition, in the IMC data the distribution of major immune cell lineages within the CD45^+^ compartment of the first trimester consisted of ∼40% NK cells and ∼1% (T cells). Importantly, our data are not contradicting current dogmas, because consistent with literature we observed decreased NK cell frequencies and increased T cell frequencies throughout gestation (Vince et al., 1990; Starkey et al., 1988; Van Der Zwan et al., 2020; Williams et al., 2009). In addition, our IMC data are in line with previous studies using immunohistochemical staining to determine the distribution of decidual leukocyte populations throughout gestation (Williams et al., 2009; Haller et al., 1993).

By combining multiple myeloid markers (e.g. CD14, CD68, CD163, CD11b), our IMC panel was well suited to accurately determine the frequency of the myeloid population within the CD45^+^ compartment. In addition, the quantification strategy we used in our analysis is highly accurate because all markers were measured simultaneously on the same tissue slide. We binarized our data to correct for staining intensity differences between samples caused by the time until fixation and the fixation procedure (Ijsselsteijn et al.). Moreover, we used machine learning methods to identify cells, instead of manually counting cells or by using subjective grading strategies (Hamilton et al., 2012). For the microenvironment analysis, we compared different pixel distances between cells. We chose a 10-pixels distance between cells because it most optimally represented the direct cellular microenvironment: the first cells adjacent to the cell of interest were identified without another cell in-between, meaning that two neighboring cells may directly affect each other by contact and/or by secreted products. Furthermore, we confirmed by permutation testing that the interactions we found do not occur at random.

Within the myeloid compartment (excluding granulocytes), we identified six phenotypically distinct subclusters. We detected the presence of a CD14^+^CD56^+^ (dMØ3) subcluster, which has not previously been described in the decidua. Upon unsupervised clustering, these cells cluster together with the myeloid cell compartment, rather than the NK cell compartment. They are positive for NK cell markers CD56 and CD7, and for the myeloid markers CD68, CD4, CD163, CD204, and DC-SIGN. In addition, some cells are positive for NKG2A and CD11c, which can be present on both NK cells and myeloid cells. Furthermore, they are positive for the activation markers CD39, TIM3, and CD38. This cluster does not seem to directly interact with trophoblasts. Upon cross referencing we also identified this subcluster in our previous SMC data (Van Der Zwan et al., 2020), and in the first trimester scRNAseq dataset from Vento-Tormo et al. (2018), where these cells similarly cluster together with other myeloid cells rather than NK cells. Currently, there is limited information on CD14^+^CD56^+^ cells. Some studies showed that progenitors expressing myeloid markers can develop into NK-like cells (MáRquez et al., 1998; Perez et al., 2003; Grzywacz et al., 2011). Furthermore, Sconocchia et al. found CD14^+^CD56^+^HLA-DR^+^CD11b^+^CD33^+^ cells in peripheral blood from healthy individuals that were capable of inducing more T cell proliferation than their CD56^−^ counterpart (Sconocchia et al., 2005). Furthermore, they produce detectable levels of IL-6 and IL-1β besides the typical monocyte cytokines *in vitro* (Sconocchia et al., 2005). Future functional analyses of the decidual CD14^+^CD56^+^ population may help to point out more specifically its function in the placenta.

The two dominant myeloid subclusters in first trimester were lacking expression of HLA-DR (dMØ1, dMØ4). Based on the reduced expression of several markers besides HLA-DR (e.g. CD45, CD4, NKG2A, CD38, and CD39), these cells likely have a different activation status than the HLA-DR^+^ subclusters (Pfau et al., 2000; Zhen et al., 2014; Maeda et al., 2013; Amici et al., 2018; Cohen et al., 2013). It has been described that most myeloid cells in the decidua are HLA-DR positive (Ning et al., 2016; Vince et al., 1990; Bulmer and Johnson, 1984), and are thereby capable of presenting antigen to CD4^+^T cells. The HLA-DR^-^ cells appear to represent a genuine population, as we observed CD14^+^ subsets that abundantly expressed HLA-DR in the same tissue slides, excluding the possibility of a technical error. To substantiate our results, we confirmed by immunofluorescence that indeed considerable numbers of decidual myeloid cells can be detected having no or diminished surface expression of HLA-DR. Interestingly, the two HLA-DR^-^ subclusters represented the only myeloid subset to be highly present in the microenvironment of first trimester trophoblasts. This may suggest that HLA-DR^-^ myeloid cells play a role in trophoblast invasion during early pregnancy. In support of this notion, Shimada et al. described in a study on first trimester decidua samples from electively terminated pregnancies and from miscarriages with normal or abnormal fetal chromosomes that CD68^+^CD163^+^HLA-DR^-^ macrophages favored the maintenance of pregnancy at an early stage (Shimada et al., 2018). In concert with the notion that HLA-DR^-^ myeloid cells (expressing low levels of activation markers) may be involved in trophoblast invasion, it has been shown that activated macrophages can inhibit trophoblast invasiveness *in vitro*, which might be disadvantageous during early placentation (Bauer et al., 2004; Renaud et al., 2005). *In vitro* analysis of the two HLA-DR^-^ subclusters would need to point out how they may influence trophoblast invasion, and whether trophoblasts and/or their secreted products may influence myeloid cell phenotype and function.

The role of myeloid cells at term appears to be different compared to first trimester, as there no longer is intimate contact with trophoblasts, and their immune cell microenvironment has changed. In first trimester, we detected mostly dNK1a and dNK1b NK subclusters in the microenvironment of all myeloid subclusters. Thereby we strengthen the current hypothesis that dNK1 represents the main NK cell subset that is important for spiral artery remodeling in first trimester (Vento-Tormo et al., 2018). Interestingly, both HLA-DR^-^ subclusters, as well as the dNK1a NK subcluster, were highly abundant in the microenvironment of trophoblasts during first trimester. Further studies need to point out if these cells work in harmony and if disturbance of either one is related to improper trophoblast invasion. As expected, during gestation the microenvironment of the myeloid subclusters changed from being NK cell-rich to being T cell-rich.

When evaluating the microenvironment of trophoblasts it is clear that there are less immune cells in their vicinity at term compared to first trimester. This decrease is likely caused by the production of fibrinoid tissue secreted by the trophoblasts, whereby they embed themselves. The matrix-type fibrinoid produced by the trophoblast cells is thought to be needed to anchor the placenta to the uterine wall, to prevent bleedings when the placenta detaches at delivery, and to regulate trophoblast invasion by cell surface integrins (Kaufmann et al., 1996). Integrins are important in regulating all aspects of immune cell functioning, both when maintaining homeostasis and during inflammation and regulation (Harjunpää et al., 2019). From the perspective of the trophoblasts the matrix-like fibrinoid may create a physical barrier that helps prevent allo-recognition by immune cells. Furthermore, it may help limiting the invasion by trophoblasts to go any further at a time point where implantation and spiral artery remodeling have already been achieved.

The IMC technique has low throughput and is suitable as an explorative approach to generate new concepts. Hence, one of the limitations of this study is the small sample size, which also limited the statistical analyses. For first trimester we have included 3 samples. Therefore, we performed statistical testing on the cluster frequencies and found that the differences (median distance) between 1^st^ trimester samples (3.51) is smaller than 1^st^ trimester against 2^nd^ trimester (5.43) or term samples (5.55) (data not shown). Next to that, we observed a clear difference to the interaction profiles in the different trimesters and therefore did not increase the sample size. Despite the limited sample size the immune cell distribution was relatively homogeneous overall samples of the different trimesters, except for one term sample (2398). This sample did not reveal many immune cells in its microenvironment, possibly because of the relative low frequency of immune cells compared to trophoblasts (ratio: 0.311) present in this sample compared to other samples. Furthermore, findings of kinetics for cell types over time were in concordance with previous literature showing that NK cell frequencies decreased, and T cell frequencies increased throughout gestation. In addition, we added an extra layer of information by in-depth immune profiling combined with the study of spatial cell orientation. It needs to be mentioned that cells found to be in each other’s microenvironment do not necessarily have interaction. Hence, further functional analyses will need to be performed to determine the exact role of each cell in its microenvironment and by what mode it is affecting any surrounding cells.

In summary, we phenotypically determined immune cell subclusters and trophoblasts present in the decidua throughout different trimesters and studied their microenvironment. During healthy pregnancy, myeloid cells show a high abundance at the maternal-fetal interface. At the three trimesters of gestation these cells are dynamic in their phenotype and interactions. In future studies, the results can be used to set up substantiated *in vitro* culture experiments to identify the consequences of cell interactions as found in the current study. Furthermore, once the role of specific maternal decidual immune cells is clear in healthy pregnancy, any deviations in their characteristics may give insight in the role they play in pregnancy complications.

### Limitation of the study

Here we visualize the spatial orientation of maternal immune cells and fetal trophoblasts within the maternal-fetal interface, however we did not study the maternal stromal cells, which are also present in high numbers as can be observed in Figure S5A. When making the single cell mask that is necessary to analyze that many different markers simultaneously, the nucleus of the cells needs to be visible. When cutting 4-μm slides, cells with large cytoplasm might lead to a gap in the mask and these cell interactions will be missed in the microenvironment analysis. This could possibly lead to an underestimation of the number of interactions of cells with large cytoplasm (e.g., macrophages and trophoblasts). Furthermore, the microenvironment analysis is limited to one cell next to another and does not include multiple different cells surrounding one cell, which would give a more complete image of the microenvironment. However, this type of analysis remains challenging to perform as this IMC technique is relatively new and analysis techniques are still under development.

This study is broad, focusing on and giving an overview on many different cell’s microenvironments. Therefore, no functional test was performed because that might take away the overview purpose of the study. Because of this we can only speculate on the effect that cells in each other’s microenvironment may have. Future studies with functional testing may address currently forwarded hypotheses.

## STAR★Methods

### Key resources table

**Table:** 

REAGENT or RESOURCE	SOURCE	IDENTIFIER
**Antibodies**		

CyTOF antibodies in Table 2.	In Table 2 of this paper	In Table 2 of this paper
Collagen IV (Dako, M0785),	Dako	M0785
Fibronectin (Sigma-Aldrich, F3648)	Sigma-Aldrich	F3648
Laminin (Sigma-Aldrich, L9393).	Sigma-Aldrich	L9393
goat-anti-mouse or rabbit Ig-HRP (Dako, K500711)	Dako	K500711
goat anti-rabbit IgG-AF546 (Invitrogen, A-11035)	Invitrogen	A-11035
goat anti-mouse IgG1-AF488 (Invitrogen, A-21121)	Invitrogen	A-21121

**Chemicals, and peptides**		

H_2_O_2_ (Merck, 107209).	Merck	107209
3,3′-Diaminobenzidine (Dako, K5007)	Dako	K5007
xylene (VWR, 28975.291)	VWR	28975.291
ethanol (Merck, 1009831000)	Merck	1009831000
citrate buffer (BioLegend, 420902)	BioLegend	420902
superblock (ThermoScientific, 37580)	ThermoScientific	37580
Hematoxylin Sigma-Aldrich, 51275	Sigma-Aldrich	51275
DAPI-ProLong Gold (Invitrogen, P36941)	Invitrogen	P36941
mounting medium (VWR, 3801731)	VWR	3801731
Iridium (125 μM)	Fluidigm	201192A

**Deposited data**		

Raw MCD files	This paper	https://data.mendeley.com/datasets/gs2bj33r6f/draft?a=4e897e07-74d4-493a-b145-9cf00e2c2a33

**Software and algorithms**		

Ilastik (v1.3.3)		https://www.ilastik.org/
Cellprofiler (v2.3.1),		https://cellprofiler.org/
ImaCytE		https://github.com/biovault/ImaCytE
Graphpad Prism (V8)		https://www.graphpad.com/scientific-software/prism/
MCD viewer		https://www.fluidigm.com/products-services/software

**Other**		

glass slides (Superfrost plus)	VWR	631–9483

### Resource availability

#### Lead contact

Further information and requests for resources and reagents should be directed to and will be fulfilled by the lead contact, Michael Eikmans (M.Eikmans@lumc.nl).

#### Materials availability

This study did not generate new unique reagents. Commercially available antibodies were conjugated to heavy metal isotopes and could be made available upon request (Table 2).

### Experimental model and subject details

Placental samples were obtained from elective abortions of first (n = 3) and second trimester (n = 5), primary c-sections (n = 2), secondary c-sections (n = 1) and spontaneous delivery (n = 2) with informed consent (Table 1). The study was carried out according to the guidelines issued by the Medical Ethics Committee of the Leiden University Medical Center (LUMC; protocols P08.087 and P11.196), and in accordance with the Declaration of Helsinki. Tonsil samples were used as controls for IMC staining and taken along in every staining to check the consistency in antibody labeling (data not shown). Tonsil samples were obtained from the department of Pathology of the LUMC and were anonymized and handled according to the medical ethical guidelines described in the Code of Conduct for Proper Secondary Use of Human Tissue of the Dutch Federation of Biomedical Scientific Societies. All tissues were cut into 4-μm sections and placed on glass slide.

### Method details

#### Immunohistochemistry and immunofluorescence

##### Deparaffinization of tissues

Slides were rinsed three times in xylene for 5 minutes, two times in 100% ethanol for 5 minutes, once in 70% and 50% ethanol for 5 minutes.

##### Heat induced epitope retrieval

Slides were added to pre-heated 10mM citrate buffer and microwaved for 10 minutes (100 watt). Excess buffer was removed and with the slides in the buffer allowed to cool down for 30 minutes. Slides were place on a tray and blocked with 200 μL superblock for 30 minutes.

##### Dehydration

After the staining dip the slide in 50% ethanol, 70% ethanol and 2x in 100% ethanol. Dry slides with air pump and rinse 3x for 1′ with xylene.

##### H&E staining

Deparaffinized tissue slides were washed with MQ for 2 minutes and stained with Mayer’s hematoxylin for 4’. Next, they were washed under running tap-water for 5′ and stained with Eosin for 1’. Lastly, they were dehydrated and covered using mounting medium and a coverslip. Consecutive slides were used for IMC staining within 24 hours.

##### Immunohistochemistry stainings

Slides of three term samples were stained for Collagen IV, Fibronectin or Laminin. The slides were first deparaffinized and heat induced epitope retrieved, then incubated for 20 minutes in 0.3% H_2_O_2_. 100 μL of the primary antibodies were added to the slide. After 1 hour incubation on room temperature slides were washed 3x for 5′ with PBS after which the slides were incubated for 1 hour with goat-anti-mouse or rabbit Ig-HRP and again washed 3x for 5′ with PBS. Signal was visualized by incubation for 5′ with 3,3′-Diaminobenzidine as a chromogen after the slides were washed again 3x for 5′ with PBS. Lastly the slides were 20 seconds counterstained with hematoxylin and washed for 5′ in tap water. Lastly the slides were dehydrated and covered using mounting medium and a coverslip.

##### Immunofluorescence stainings

For validation of HLA-DR expression or absence on myeloid cells immunofluorescence (IF) staining was performed, with HLA-DR (clone in Table 2) and CD14 (clone in Table 2). After tapping of the excess blocking solution, 100 μL of the diluted primary antibodies were added to the slide and incubated for 1 hour at room temperature. The slides were washed 3x for 5′ with PBS. Next, the slides were incubated for 1 hour at room temperature in the dark with IF goat anti-rabbit IgG-AF546 and goat anti-mouse IgG1-AF488 to visualize the primary antibodies before the slides were dehydrated and covered using DAPI-Pro-Long Gold and a coverslip.

#### Imaging mass cytometry

##### Mass cytometry antibodies

Heavy metal isotope-tagged monoclonal antibodies are listed in Table 2. All, but keratin, α-smooth muscle actin (SMA), CD4, EGF-R, Vimentin, and HLA-G antibodies were conjugated with heavy metal isotopes in-house using the MaxPar X8 Polymere Antibody Labeling Kit according to the manufacturer’s protocol (Fluidigm, California, USA). EGF-R was pre-conjugated by Fluidigm. Conjugation of two keratin antibody clones to 106Pd, was performed using a protocol adapted from Schulz et al. (Schulz and Mei, 2019). Conjugation with 209Bito α-SMA was performed using a protocol adapted from Spitzer et al. (2017). Cisplatin 194 and 198 were conjugated to Vimentin and HLA-G using a protocol adapted from Mei et al. (2016). CD4 was stained using a secondary staining step with α-mouse-145Gd. All 43 primary antibodies and the one secondary antibody were titrated to determine the optimal labelling concentration. Additionally, all antibodies unconjugated and conjugated to a metal were tested by immunohistochemistry before being used in IMC as described above. For each of the 42 metal-tagged primary antibodies the best of two incubation options was used: 5 hours at room temperature or overnight at 4°C.

##### Imaging mass cytometry staining

Imaging mass cytometry antibody staining was performed as previously described by Ijsselsteijn et al. (Ijsselsteijn et al. (2019). First excess superblock solution was taped of the slides after the deparaffinization, and heat induced epitope retrieval. Next, 100 μL of anti-CD4 (mouse IgG1, dilution in Table 2) diluted in staining buffer (PBS/1%BSA/0.05%Tween) was added to the slides and incubated overnight at 4°C. After washing 3x for 5′ with staining buffer, the sections were incubated with 100 μL secondary anti-mouse-145Gd antibody for 1 hour at room temperature. The sections were washed again 3x for 5′ with staining buffer and incubated with the metal-labeled antibodies for 5 hours at room temperature (Table 2). After washing 3x for 5′, the slides were then incubated with the second antibody mix for overnight incubation at 4°C. After washing 3x for 5′ the slides were incubated 5′ with 100 μL Iridium (1.25 μM) nuclear staining. Lastly, the slides were washed 2x for 5′ with staining buffer an 1x for 5′ with demineralized water, and dried under an air flow.

##### Imaging mass cytometry data acquisition

The Hyperion was autotuned, using a three-element tuning slide according to the manufacturer’s protocol (Fluidigm). Using the consecutive HE-stained slides the Regions of interest (ROIs) (areas varied from 0.26 mm^2^ to 1.84 mm^2^) on the IMC slides could accurately be set on the decidua. This resulted in five to twelve ROIs per placenta sample. The selected ROIs were ablated at 200 Hz.

##### Data analysis

###### Creating a single cell mask using cell segmentation

For each ROI a single cell mask was created as previously described, with some adjustments (Ijsselsteijn et al.,2021). First, based on the consecutive H&E staining and IMC trophoblast markers the villi and extravillous space were removed from all ROIs in the exported DNA file to analyze the decidua basalis only. Ilastik (v1.3.3) was used to create three probability maps based on the DNA signal (193^Iridium^), myeloid markers (CD14, CD68, and CD163), other immune cells (CD45 and CD56). These three probability maps were together loaded in Cellprofiler (v2.3.1), with DNA as primary object, their size was increased by 2 pixels (2 μm) to make sure membrane marker expression was included in all types of cells and myeloid markers and other immune cells were loaded as secondary object. Then a single cell mask per ROI was from Cellprofiler. For each ROI the mask was compared to the original IMC data (as in Figure S6C). Trophoblast- and maternal stromal cell markers were not used to create the mask because of the large cytoplasm size. Therefore, the signal often does not belong to a nucleus or is too close to other nuclei to mask correctly. Because all objects (DNA signal) in the mask were expanded by two pixels, membrane staining besides nuclear staining was included for all types of cells.

###### Background removal and data normalization

To improve data recovery and reduce noise, semi-automated thresholding by a machine learning algorithm in Ilastik was used to separate true signals from noise as was described earlier (Berg et al., 2019; Ijsselsteijn et al., 2021). For each individual marker, the algorithm was trained to separate noise from signal which resulted in binary pixel values.

###### Phenotyping of segmented cells

All masks together with all binarized thresholded ROIs were loaded in ImaCytE (Somarakis et al., 2021). Each cell in the mask was combined with its corresponding thresholded pixel intensity file, and FCS files could be exported with the marker expression per cell as relative frequency of positive pixels. These single-cell FCS files were analyzed by t-SNE in Cytosplore (v2.3.1). Seven t-SNEs were performed. First a t-SNE was made on absence or presence of marker expression, where a cluster with no marker expression (background1) and a cluster with marker expression was determined. On the cells with marker expression another t-SNE was performed where immune cells were separated from other tissue cells (Figure S3). Next a separate tSNE was made on immune cells and on other tissue cells. For tissue cells a distinction could be made between trophoblasts, maternal stromal cells, and unknown cells with not enough markers to phenotype precisely. For the immune cells four major immune cell lineages could be identified, namely myeloid cells, NK cells, granulocytes, T cells, and unknown, with the latter not having enough positive markers to phenotype accurately. Another tSNE was performed on the myeloid cells, NK cells, and T cells to determine smaller subclusters (minimum of 100 cells per cluster). All cell subclusters can be found in Figure 2A and tSNE clustering in Figure S3.

###### Comparing SCM and IMC data

SCM and IMC data were compared by using Cytosplore. Both single cell FCS files were loaded separately in Cytosplore after which CD45 positive and DNA positive cells were selected (54.351 cells for IMC, 7.198.273 cells for IMC (down sampled to 54.351 cells for visualization)). Next, the 17 overlapping markers between panels (CD45, CD14, CD15, CD69, CD4, CD8a, CD163, CD103, CD11c, HLA-DR, CD45RA, CD3, CD38, CD45RO, PD1, CD56 and CD11b) were used to generate major immune lineage clusters. Frequencies were exported and analyzed in Graphpad Prism (V8).

### Quantification and statistical analysis

#### Microenvironment analyses

The assigned phenotypes were loaded back into ImaCytE with the masks to localize the phenotypes in the tissue (Somarakis et al., 2021)**.** The microenvironment analysis was done per sample, combining all ROIs per sample (Table 1). The distance (in pixels) that determines cell proximity was set to 10 (1 pixel = 1 μm^2^). We found with this setting that the first cells surrounding the cell of interest were identified without another cell in-between, meaning that two neighboring cells may directly affect each other by contact and/or by secreted products (Figure S6A, S6B and S6C). Next, the microenvironments per phenotype were exported per sample for further data analysis including permutation z-scores. The percentages of the microenvironment of each cell with all other cells were calculated per sample by using the count of at least one co-localized cell in a 10-pixel radius divided by the absolute number of cells in that cluster of that sample (observed microenvironment). Next, the frequency of the immune cell clusters was calculated (within the total of all immune cells and trophoblast cells). These percentages were used to determine the chance of random cells in the microenvironment. This was done by multiplying the frequency of the cell cluster of interest with the frequency of the cell cluster it could interact with. The expected percentage was subtracted from the percentage of the observed microenvironment (Figure S6D). For data visualization, heatmaps show z-scores per row based on the percentages of the corrected microenvironment analysis. The z-score shows the amount of SD the sample value is above or below the mean of the specific row. Other graphs show percentages of the corrected microenvironmental analysis data. Permutation z-scores were used to confirm that the calculated interactions we find do not occur at random. A permutation z-score of 1.96 (probability of <0.05) was used as cutoff.

#### Statistics

IMC and SMC data ware plotted in box-and-whiskers plots visualizing min to max points using GraphPad Prism (V8). The two groups were compared by Mann-Whitney test corrected for the three trimesters by Bonferroni correction. The microenvironment analyses were visualized by their median and interquartile range. When first, second, and third trimester were compared, the Kruskal-Wallis test and Dunn’s multiple comparisons test were used. When multiple clusters were compared over the same trimester, the Friedman test and Dunn’s multiple comparisons test were used.

## Data Availability

• MCD data files have been deposited at Mendeley Data and are publicly available as of the date of publication, with accession number https://data.mendeley.com/datasets/gs2bj33r6f/draft?a=4e897e07-74d4-493a-b145-9cf00e2c2a33 • This paper does not report original code. • Any additional information required to reanalyze the data reported in this paper is available from the lead contact on request.
