# Supplementary material for: Imaging mass cytometry reveals the prominent role of myeloid cells at the maternal-fetal interface
Source: iScience. 2022 Jun 20;25(7):104648. doi: 10.1016/j.isci.2022.104648 (PMC9257341; doi:10.1016/j.isci.2022.104648)
Supplement: Document S1. Figures S1–S7 [file mmc1.pdf]

iScience, Volume *25*

Supplemental information

Imaging mass cytometry reveals the prominent role of myeloid cells at the maternal-fetal interface

Juliette Krop, Anita van der Zwan, Marieke E. Ijsselsteijn, Hanneke Kapsenberg, Sietse J. Luk, Sanne H. Hendriks, Carin van der Keur, Lotte J. Verleng, Antonis Somarakis, Lotte van der Meeren, Geert Haasnoot, Manon Bos, Noel F.C.C. de Miranda, Susana M. Chuva de Sousa Lopes, Marie-Louise P. van der Hoorn, Frits Koning, Frans H.J. Claas, Sebastiaan Heidt, and Michael Eikmans

Supplementary Figure S1

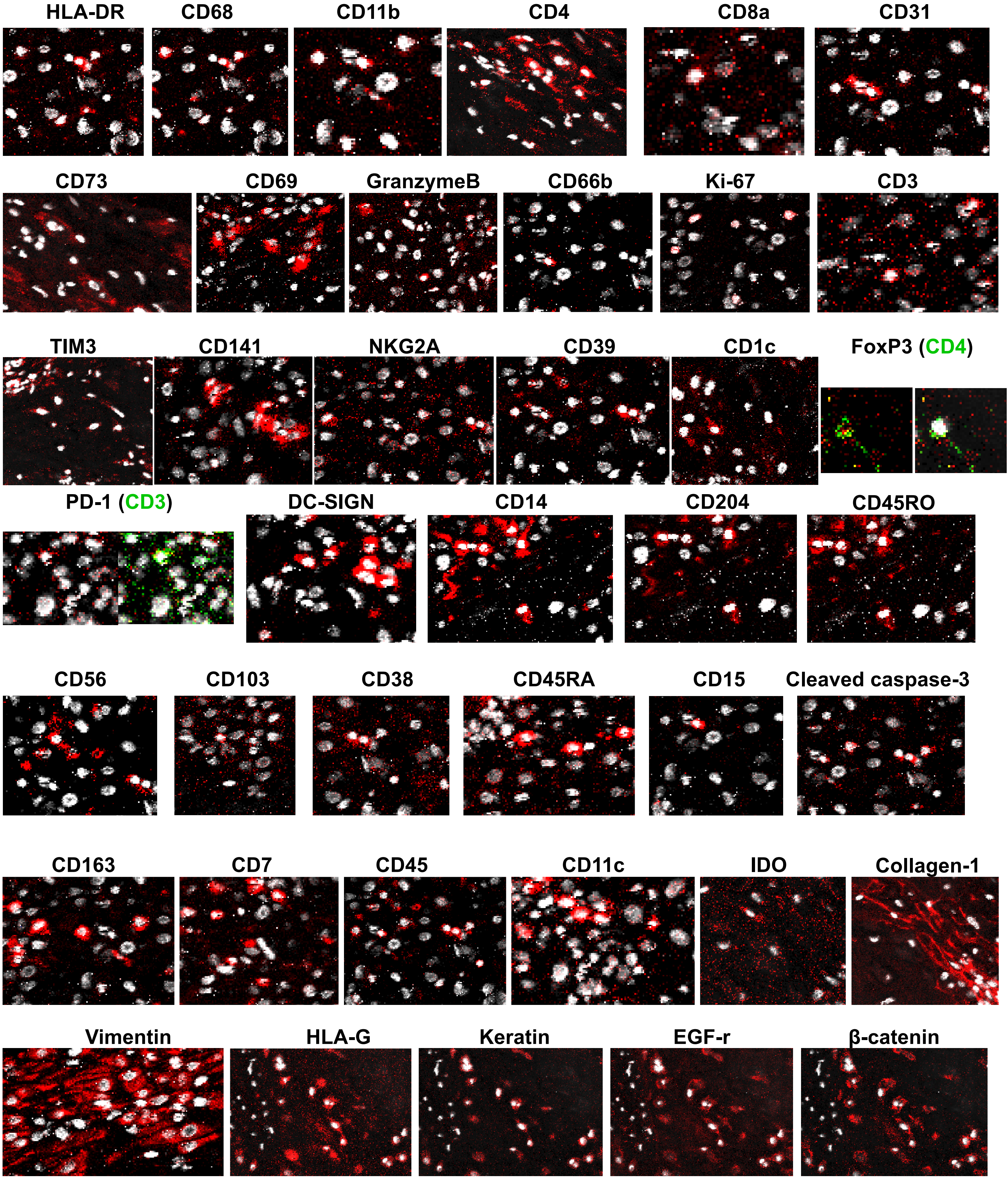

Supplementary Figure S2

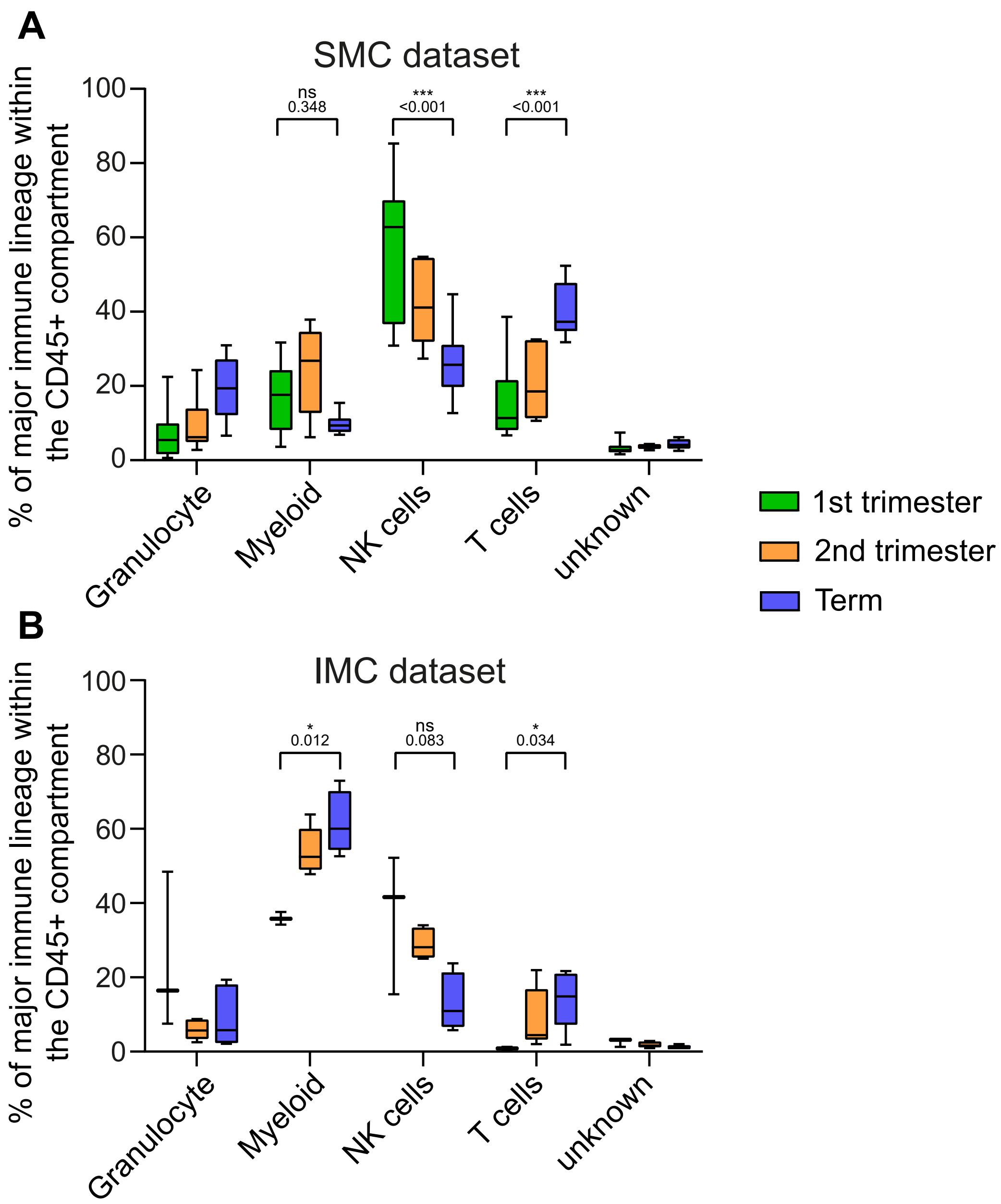

Supplementary Figure S3

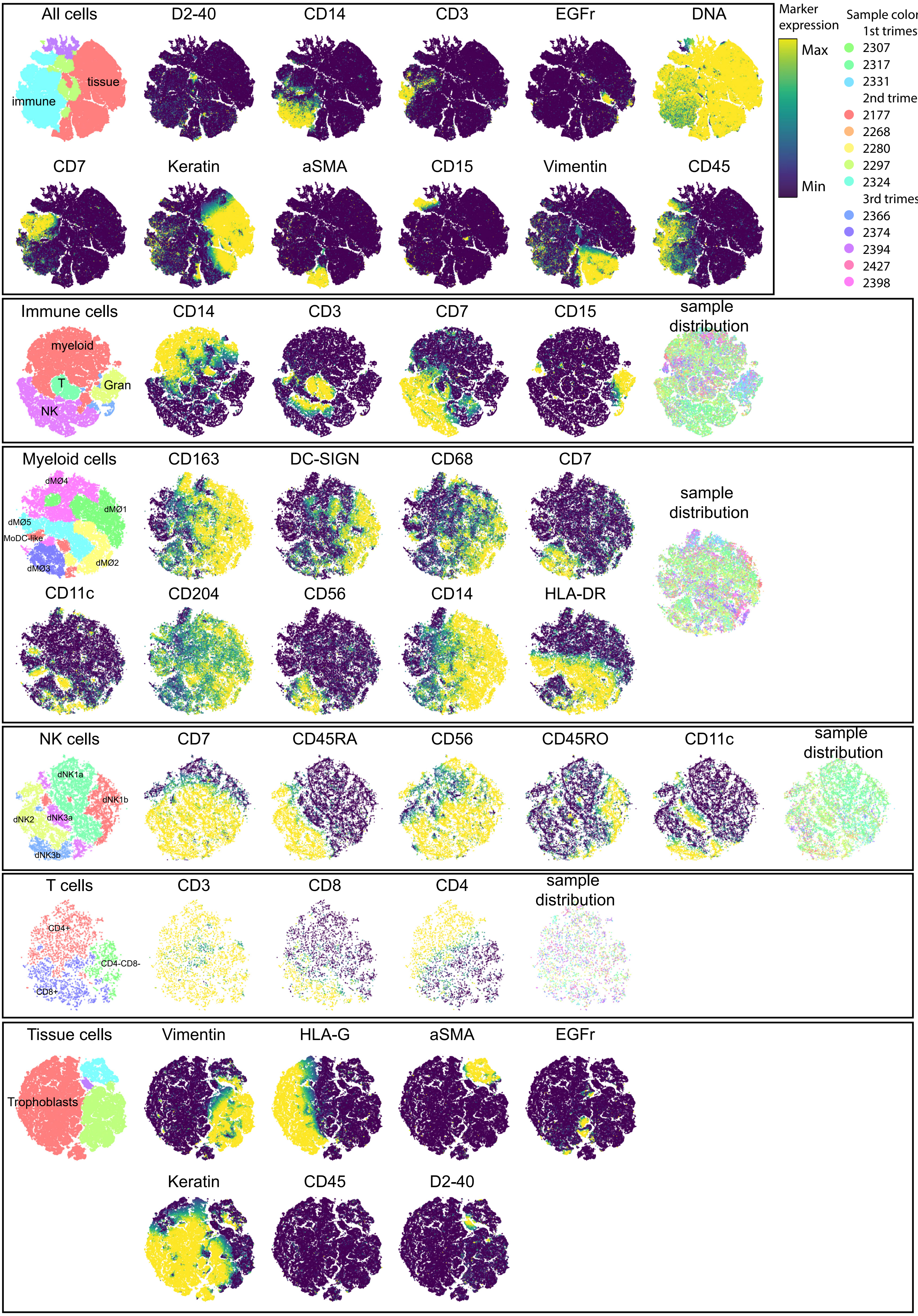

Supplementary Figure S4

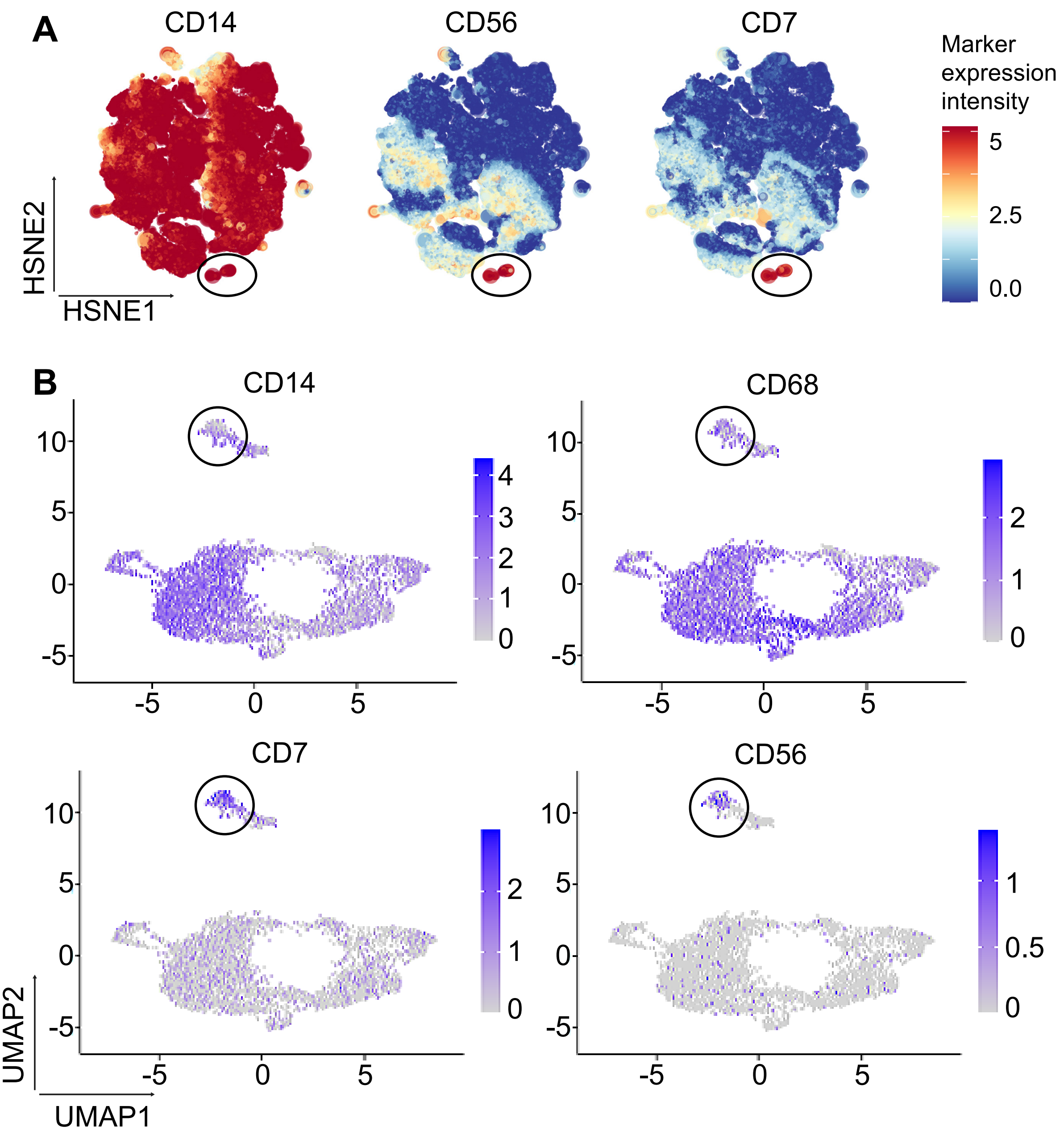

Supplementary Figure S5

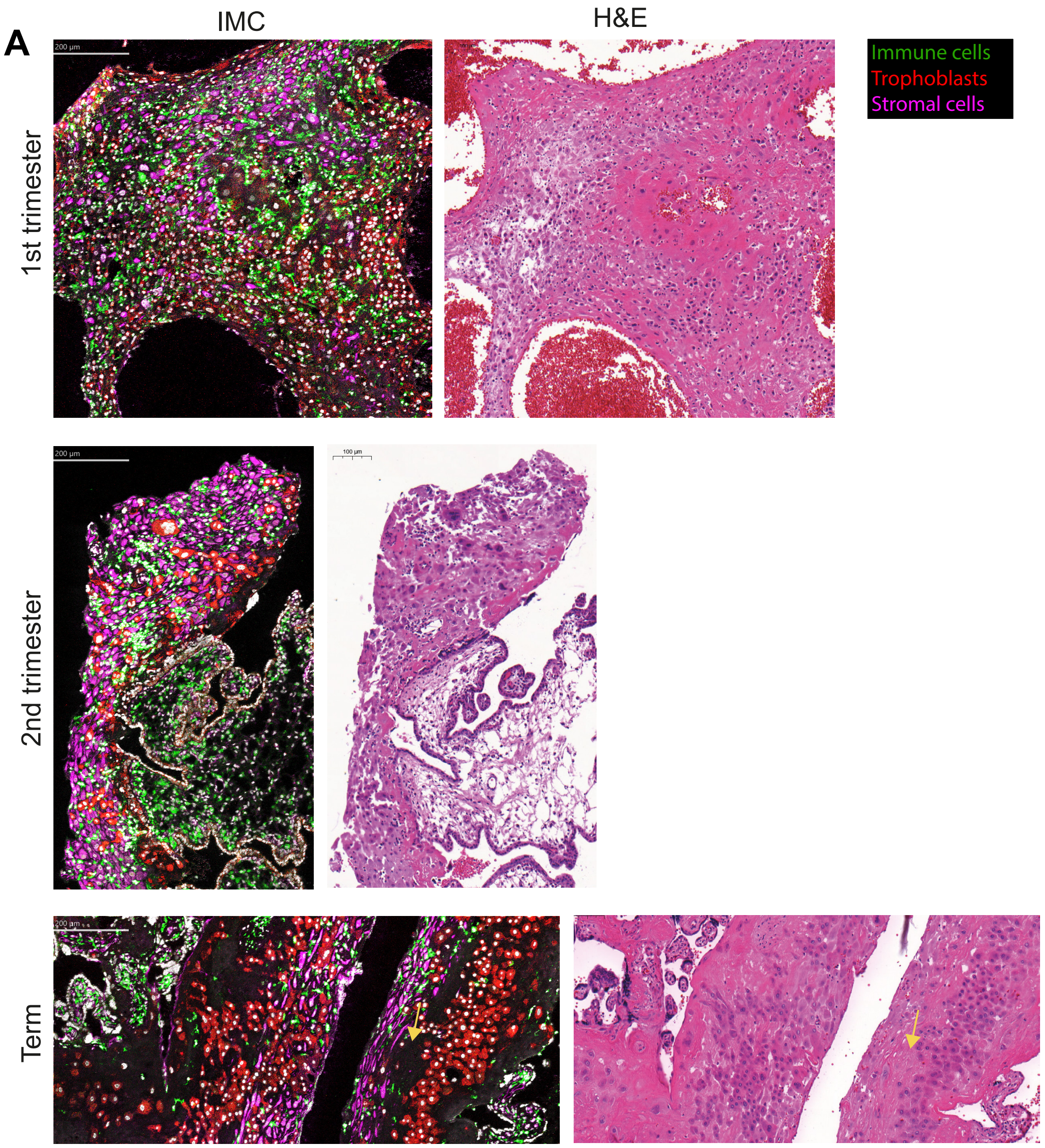

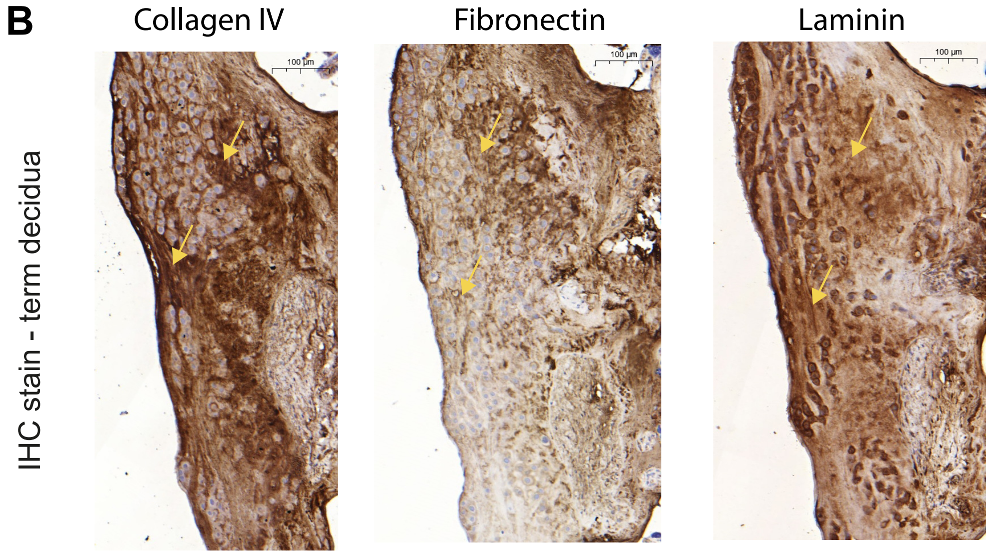

Supplementary Figure S6

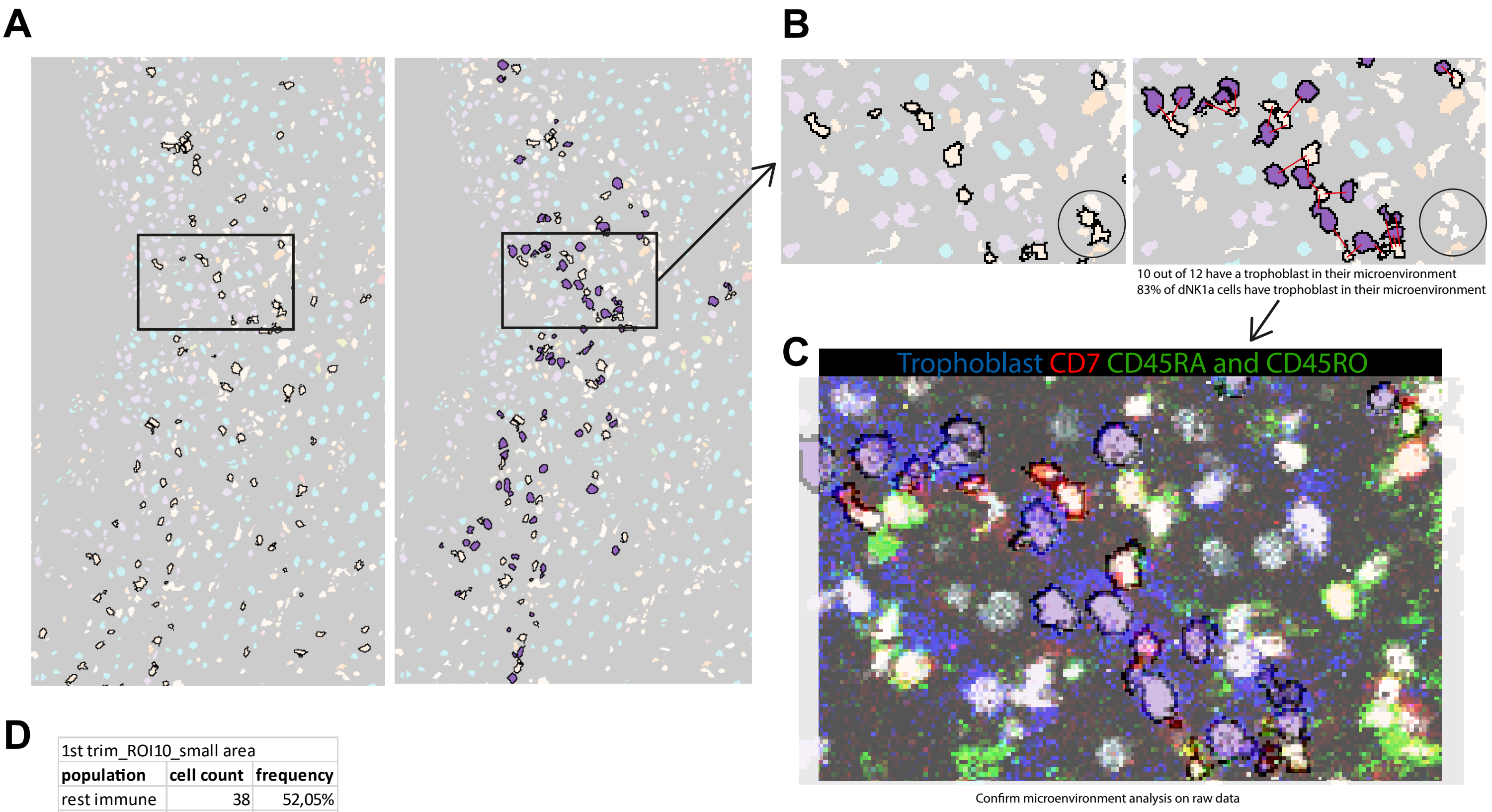

D

1st trim_ROI10_small area		
**population**	**cell count**	**frequency**
rest immune	38	52,05%
dNK1a	12	16,44%
Trophoblast	25	34,25%

1st trim_ROI10_small area						
**Phenotype of interest**	**Phenotype of microenvironment**	**# of at least one colocalized cell**	**# of celltype of interest**	**% observed intereaction**	**% expected interaction**	**% interaction**
dNK1a	Trophoblast	10	12	83,33%	5,63%	77,70%

Supplementary Figure S7

A

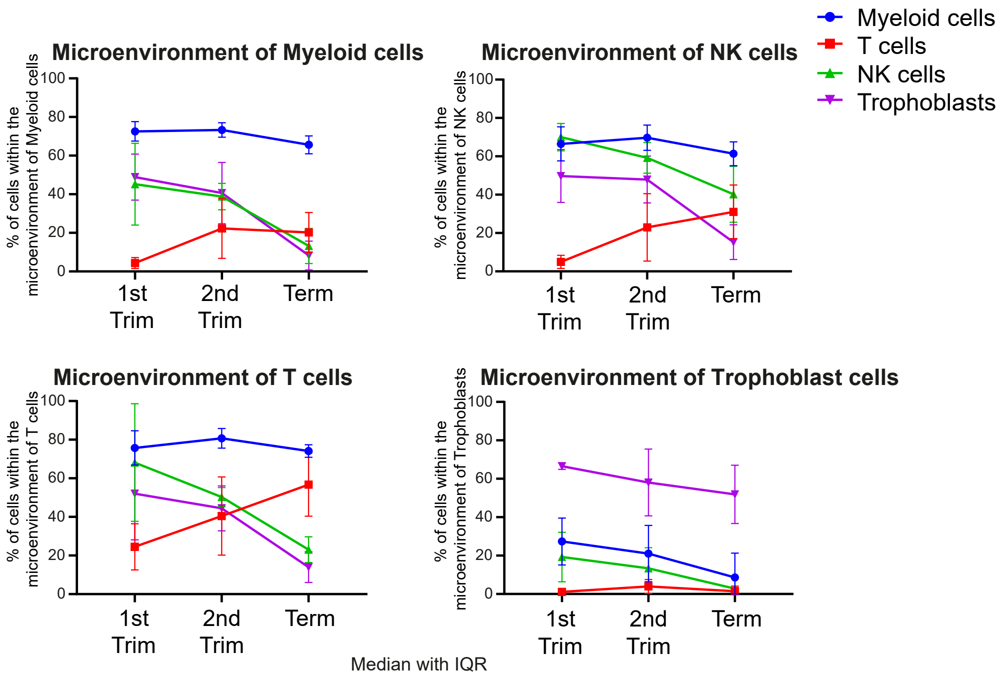

B

Permutation testing major immune lineages

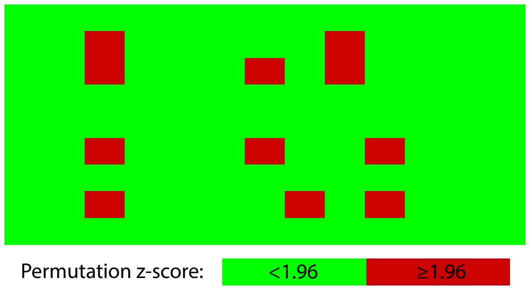

Cell of interest	*Cell in Microenvironment*
Myeloid cells	Myeloid cells
Myeloid cells	T cells
Myeloid cells	NK cells
T cells	Myeloid cells
T cells	T cells
T cells	NK cells
NK cells	Myeloid cells
NK cells	T cells
NK cells	NK cells

Figure S1. Marker expression of each marker used in IMC. Related to Table 1.

Visualization of all 42 markers in the IMC panel, exported from MCD viewer. Markers were individually thresholded for improved visualization. Marker expression is depicted by a red signal, DNA expression by a white signal. FoxP3 is hard to visualize with DNA and therefore is visualized with CD4 (green) co-expression. PD-1 is a dim marker and visualized with CD3 (green) co-expression.

Figure S2. Comparison of major immune cell lineages by IMC and SMC. Related to Figure 1.

Frequency of major immune cell lineages including granulocytes and unassigned cells (unknown) in the $CD45^{+}$ compartment by both SMC and IMC in first and second trimester and term samples. Data are represented as Min to Max boxplots, Kruskall-Wallis and Dunn's multiple comparisons test, *P < 0.05; **P < 0.01; ***P < 0.001.

Figure S3. tSNE plots visualizing clusters and individual marker expression. Related to Figure 2.

tSNE visualization of all clusters identified by IMC using all 42 markers in the panel for clustering. Clusters contain at least 100 cells, if during automatic clustering a cluster is smaller than 100 cells it is merged with the most similar cluster according to the dendogram created by the stochastic neighborhood analysis. Clusters only differing from dim to bright marker expression are merged, since dim and bright expression, due to previous binarization of signal intensity, is irrelevant.

Figure S4. dMØ3 population found in SMC dataset and single cell RNA sequencing dataset. Related to Figure 2.

Identification of the dMØ3 cluster (circled) in (A) suspension mass cytometry dataset, and (B) single cell RNA sequencing dataset. HSNE, Hierarchical Stochastic Neighbor Embedding; UMAP, Uniform Manifold Approximation and Projection.

Figure S5. Representative overview IMC image of trophoblasts, decidual stromal cells, and immune cells in all three trimesters. Related to figure 2.

(A) Overview image from raw data from MCD viewer of first and second trimester and term samples. DNA is visualized in white; trophoblasts are visualized in red using Keratin and HLA-G; decidual stromal cells are visualized in magenta using vimentin; and immune cells are visualized in green using CD45 and CD14. Consecutive Haematoxylin and Eosin slides of the similar location as in A for overview purposes and for visualization of fibrinoid-like tissue, indicated by a yellow arrow. (B) IHC staining of term decidua with Collagen IV, Fibronectin and Laminin, fibrinoid-like tissue is indicated by yellow arrows.

Figure S6. Microenvironment analysis explained. Related to Figure 4 and 5.

After mask design and phenotype notation, cells were loaded back into ImaCytE where interaction analysis was performed. In this example the frequency of trophoblasts (purple) in the microenvironment of dNK1a cells (white) is calculated (small region of 1st trim placenta) including correction for cell frequency. (A) visualization of dNK1a and (B) dNK1a cells with a trophoblast in their microenvironment trophoblasts cells. (B) zooming in to our example region we observed 12 dNK1a cells. Ten of those 12 have a trophoblast in their microenvironment (circle on the two that do not) which is 83%. (C) We find this is correct when comparing to raw data. (D) We calculated the cell frequency of dNK1a (16.44%) and trophoblasts (34.25%) within one sample and corrected for that by multiplying it with one another (16.44%*34.25% = 5.63%). We subtract the expected interaction from the observed interaction giving a corrected frequency for the amount of trophoblasts in the microenvironment of dNK1a cells. This was done per sample for all cell clusters.

Figure S7. Frequency and permutation z-score of the microenvironment analysis. Related to Figure 4 and 5.

(A) Frequency of major immune lineages and trophoblasts in the microenvironment of other major immune lineages and trophoblast. Other than z-scores of (Figures 4A and 5A) the percentages of immune cells in the microenvironment of other cells can amongst others visualize the constant high frequency of myeloid cells in the microenvironment of other cells. (B) Permutation z-scores of major immune lineages interactions shows that most interactions are not at random (≥1.96; probability of <0.05).
